# Unveiling Stage-Specific Flavonoid Dynamics Underlying Drought Tolerance in Sweet Potato (*Ipomoea batatas* L.) via Integrative Transcriptomic and Metabolomic Analyses

**DOI:** 10.3390/plants14152383

**Published:** 2025-08-02

**Authors:** Tao Yin, Chaoyu Song, Huan Li, Shaoxia Wang, Wenliang Wei, Jie Meng, Qing Liu

**Affiliations:** 1 College of Resources and Environment, Qingdao Agricultural University, Qingdao 266109, China; yintao@qau.edu.cn (T.Y.); lihuancomcomcom@163.com (H.L.); wang-sx@hotmail.com (S.W.); weiwl@qau.edu.cn (W.W.); 2 Qingdao Academy of Agricultural Sciences, Qingdao 266100, China; qingdaocrop@163.com; 3 College of Landscape and Forestry, Qingdao Agricultural University, Qingdao 266109, China; mengjieqau@qau.edu.cn

**Keywords:** drought stress, transcriptomics, metabolomics, flavonoid biosynthesis, stage-specific adaptation

## Abstract

Drought stress severely limits the productivity of sweet potato (*Ipomoea batatas* L.), yet the stage-specific molecular mechanisms of its adaptation remain poorly understood. Therefore, we integrated transcriptomics and extensive targeted metabolomics analysis to investigate the drought responses of the sweet potato cultivar ‘Luoyu 11’ during the branching and tuber formation stage (DS1) and the storage root expansion stage (DS2) under controlled drought conditions (45 ± 5% field capacity). Transcriptome analysis identified 8292 and 13,509 differentially expressed genes in DS1 and DS2, respectively, compared with the well-watered control (75 ± 5% field capacity). KEGG enrichment analysis revealed the activation of plant hormone signaling, carbon metabolism, and flavonoid biosynthesis pathways, and more pronounced transcriptional changes were observed during the DS2 stage. Metabolomic analysis identified 415 differentially accumulated metabolites across the two growth periods, with flavonoids being the most abundant (accounting for 30.3% in DS1 and 23.7% in DS2), followed by amino acids and organic acids, which highlighted their roles in osmotic regulation and oxidative stress alleviation. Integrated omics analysis revealed stage-specific regulation of flavonoid biosynthesis under drought stress. Genes such as *CYP75B1* and *IF7MAT* were consistently downregulated, whereas flavonol synthase and glycosyltransferases exhibited differential expression patterns, which correlated with the selective accumulation of trifolin and luteoloside. Our findings provide novel insights into the molecular basis of drought tolerance in sweet potato and offer actionable targets for breeding and precision water management in drought-prone regions.

## 1. Introduction

As the world’s fifth-largest food crop, sweet potato (*Ipomoea batatas* L.), which is rich in carbohydrates, dietary fiber, and antioxidant compounds (e.g., β-carotene and flavonoids), plays an important role in ensuring food security [1,2,3]. Due to its extensive physiological plasticity and environmental adaptability, sweet potato is considered indispensable in agricultural production systems in arid and semi-arid areas [2]. With the intensification of global climate change, drought has become one of the major abiotic stressors limiting the yield and quality of sweet potato [4]. As a typical shallow-rooted crop, sweet potato is highly sensitive to soil moisture fluctuations. This physiological characteristic makes it particularly vulnerable to water deficits during critical growth stages, such as the branching and tuber formation stage as well as the storage root expansion stage [5]. Previous studies have shown that drought significantly inhibits photosynthetic efficiency, biomass accumulation, and secondary metabolite synthesis in sweet potato, ultimately leading to yield losses of up to 30–50% [6,7]. Therefore, in the context of global climate change, which is increasing both the frequency and intensity of droughts, understanding the molecular and metabolic mechanisms underlying sweet potato responses to drought stress is crucial for developing targeted strategies to enhance drought tolerance and maintain stable yields under water-limited conditions.

The response mechanisms of sweet potato to drought stress are complex and multifaceted. Over the past decade, numerous studies have investigated the physiological responses, metabolite changes, and metabolic pathway adjustments in sweet potato under drought stress across different growth stages [8,9,10]. Regarding physiological responses, research has focused on leaf water status, photosynthesis, and chlorophyll content. Drought has been shown to reduce leaf water potential and stomatal conductance to minimize water loss [11,12]. However, this response simultaneously limits CO_2_ uptake, thereby decreasing the photosynthetic rate [13,14]. In addition, drought often reduces chlorophyll content, further impairing photosynthetic capacity [15].

Studies on metabolic changes have mainly focused on the dynamics of osmoregulatory substances, antioxidant metabolites, and phytohormones. Under drought stress, sweet potato exhibits a reduction in the water content of functional leaves, while significantly increasing the concentrations of soluble sugars, soluble proteins, free amino acids, and proline in functional leaves, fine roots, and storage roots [16]. These changes help maintain cellular water balance under water-limited conditions [17]. Moreover, earlier onset of stress leads to a greater accumulation of these substances. Notably, the early accumulation of osmotic substances induced by drought often does not fully return to normal levels after rehydration, whereas changes occurring at later stages tend to recover more readily [9]. Drought stress also promotes the accumulation of antioxidant compounds in sweet potato leaves, including flavonoids, polyphenols, and glutathione. These metabolites help scavenge reactive oxygen species (ROS) and reduce oxidative damage [8]. In addition, drought stress alters the balance of endogenous hormones. Specifically, abscisic acid (ABA) levels are significantly elevated, while indole-3-acetic acid and gibberellin levels tend to decrease, indicating a suppression of growth hormone-responsive genes to inhibit elongation and conserve water [18].

At the level of metabolic pathways, studies have mainly focused on the ABA signaling pathway, carbon metabolic processes, and secondary metabolic processes [19]. Drought stress has been shown to activate the expression of genes involved in the ABA signaling pathway in sweet potato, thereby triggering the transcriptional activation of downstream stress-responsive genes [9,20]. Additionally, drought induces shifts in carbon assimilation and partitioning pathways, promoting the accumulation of soluble sugars for osmoregulation and shifting carbon metabolism from a growth-oriented mode to a defense- and storage-oriented mode [21]. Some studies have found that drought-sensitive sweet potato varieties downregulate genes involved in specific carbon and energy metabolism pathways under stress, such as isoquinoline alkaloid biosynthesis and nitrogen and carbon metabolism [22,23]. This may reflect reduced metabolic activity and a disruption in the redistribution of carbon flow. Secondary metabolism also plays a crucial role in drought tolerance [24]. Drought markedly activates the phenylpropanoid–flavonoid biosynthesis pathway and enhances the accumulation of antioxidant secondary metabolites, such as flavonols and anthocyanins, which function to scavenge ROS, thereby alleviating oxidative stress [25,26].

Overall, sweet potato seedlings are the most sensitive to drought stress, followed by the storage root expansion stage, whereas drought resistance tends to increase during the maturation stage. Drought stress affects sweet potato growth in a stage-dependent manner, with early-stage drought (during branching and tuber formation) and late-stage drought (during storage root expansion) potentially triggering distinct physiological and molecular alterations [27,28]. Although previous studies have explored drought-induced changes in sweet potato at the physiological, biochemical, and transcriptomic levels, a significant gap still exists regarding the interaction between transcriptomic reprogramming and metabolic adjustments across different developmental stages. Therefore, integrating transcriptomic and metabolomic approaches is essential to elucidate the coordination of gene expression with metabolic regulation under drought conditions.

Among the various secondary metabolites, flavonoids have emerged as key compounds involved in plant adaptation to abiotic stress, particularly drought [29]. These compounds function as antioxidants that scavenge ROS, protect cellular components, and modulate signal transduction pathways related to stress responses [30]. While flavonoid-mediated drought tolerance mechanisms have been extensively studied in model species such as *Arabidopsis thaliana* and *Glycine max*, their specific regulatory patterns and functional contributions in sweet potato remain largely unexplored [31,32,33]. This gap is noteworthy given that sweet potato is frequently grown in marginal areas prone to water shortages and contains naturally high levels of flavonoids such as quercetin, rutin, and luteolin [34,35]. Recent studies have suggested that flavonoid biosynthesis in sweet potato may exhibit growth-stage-specific patterns under stress, but a comprehensive multi-omics investigation is lacking [36,37]. Therefore, dissecting the dynamics of flavonoid biosynthesis at both transcript and metabolite levels is essential to understand their coordinated roles in drought adaptation, and may help identify candidate biomarkers for stress-resilient cultivars.

In this study, the sweet potato cultivar ‘Luoyu 11’, which is widely cultivated in arid and semi-arid regions, was employed to systematically analyze leaf responses to drought stress during the branching and tuber formation stage and the storage root expansion stage. We achieved this by integrating transcriptomic and broad-targeted metabolomic analyses. The objectives were as follows: (1) identify key differentially expressed genes (DEGs) and differentially accumulated metabolites (DAMs) associated with drought responses at different growth periods of sweet potato; (2) perform Kyoto Encyclopedia of Genes and Genomes (KEGG) pathway enrichment analysis to uncover major metabolic and regulatory pathways involved in the drought response; (3) conduct a comprehensive multi-omics analysis to elucidate the coordinated regulation between genes and metabolites under drought conditions. Understanding these interactions is of great significance for advancing our understanding of the molecular mechanisms underlying drought tolerance in sweet potato. It also provides a basis for identifying key regulatory pathways and potential biomarkers, thereby supporting targeted precision irrigation management strategies across different growth periods.

## 2. Results

### 2.1. Transcriptomic Analysis of Sweet Potato Leaves Under Drought Stress

**Figure 1:**
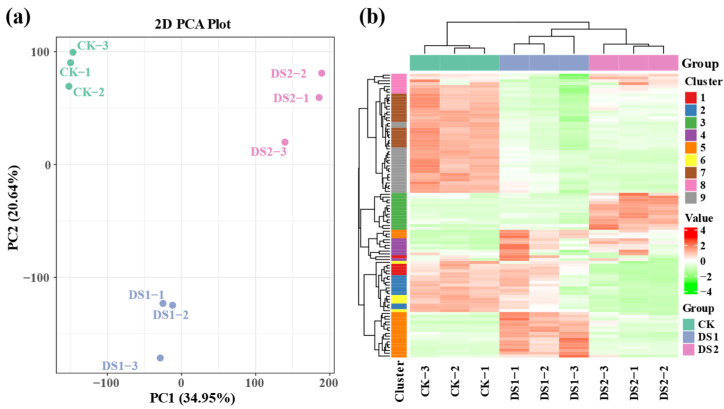
Transcriptomic analysis of sweet potato (*Ipomoea batatas* L.) leaves under drought stress at different growth stages. (**a**) Principal component analysis (PCA) showing the overall differences in gene expression profiles among treatment groups. (**b**) Hierarchical clustering heatmap displaying significantly differentially expressed genes (DEGs). Rows represent individual DEGs, columns represent individual samples. The color gradient from red (upregulated) to green (downregulated) indicates normalized gene expression levels (Z-score) relative to the mean expression across all samples. Gene clusters (1–9), identified via K-means clustering, are indicated on the left side of the heatmap. CK, with soil moisture maintained at 75 ± 5% field capacity (FC) throughout the growth period; DS1, drought stress with soil moisture maintained at 45 ± 5% FC for 15 days during the branching and tuber formation stage (55–70 days post-planting); DS2, drought stress with soil moisture maintained at 45 ± 5% FC for 15 days during the storage root expansion stage (90–105 days post-planting).

To investigate the overall transcriptomic responses of sweet potato leaves to drought stress at different growth stages, Principal component analysis (PCA) was conducted on the gene expression profiles across all treatment groups (Figure 1a). The first two principal components (PC1 and PC2) explained 34.95% and 20.64% of the total variance, respectively. The control group (CK, with soil moisture maintained at 75 ± 5% of field capacity [FC] throughout the growth period) clustered distinctly from both drought stress treatments—DS1 (45 ± 5% FC during the branching and tuber formation stage, 55–70 days after planting) and DS2 (45 ± 5% FC during the storage root expansion stage, 90–105 days after planting)—indicating a significant transcriptional shift in response to drought stress. Moreover, the DS1 and DS2 groups formed separate clusters, suggesting stage-specific transcriptomic responses.

Hierarchical clustering analysis (HCA) was further performed on DEGs (Figure 1b). The heatmap revealed clear patterns of upregulation and downregulation across treatments. CK samples exhibited more uniform gene expression, whereas DS1 and DS2 showed pronounced shifts. Genes were grouped into nine distinct clusters via K-means clustering, reflecting shared expression trends across treatments.

### 2.2. Differentially Expressed Genes Analysis in Response to Drought Stress

**Figure 2:**
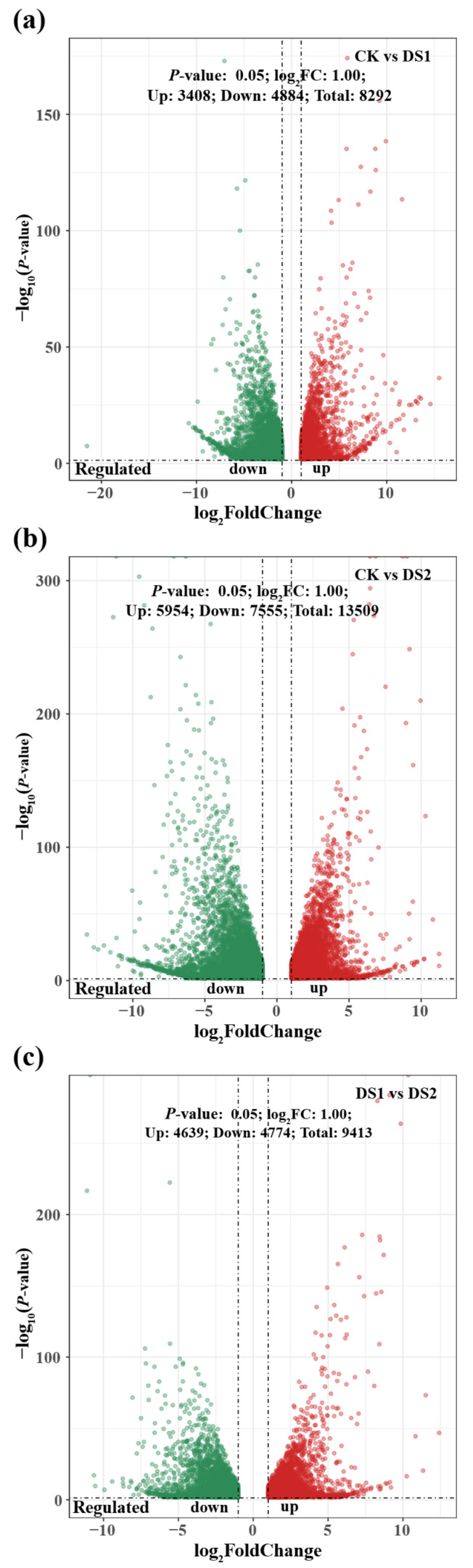
Volcano plots showing significantly differentially expressed genes (DEGs) in sweet potato (*Ipomoea batatas* L.) leaves under drought stress at different growth stages. Pairwise comparisons include: (**a**) CK (control; soil moisture maintained at 75 ± 5% field capacity (FC) throughout the growth period) vs. DS1 (drought stress; soil moisture maintained at 45 ± 5% FC for 15 days during the branching and tuber formation stage, 55–70 days after planting); (**b**) CK vs. DS2 (drought stress; soil moisture maintained at 45 ± 5% FC for 15 days during the storage root expansion stage, 90–105 days after planting); and (**c**) DS1 vs. DS2. The x–axis represents the log_2_ fold change (log_2_FC) of gene expression, and the y–axis represents statistical significance (−log_10_ *p*-value). Each dot represents a gene: red and green dots indicate significantly upregulated and downregulated genes, respectively (|log_2_FC| ≥ 1 and *p* < 0.05). Vertical dashed lines indicate the fold change threshold (log_2_FC = ±1).

Differentially expressed genes were identified through pairwise comparisons among the treatment groups (Figure 2). In the CK vs. DS1 comparison (Figure 2a), 8292 DEGs were detected, including 3408 upregulated and 4884 downregulated genes under drought stress during the branching and tuber formation stage, suggesting substantial transcriptomic reprogramming. In the CK vs. DS2 comparison (Figure 2b), representing drought stress during the storage root expansion stage, 13,509 DEGs were identified, with 5954 upregulated and 7555 downregulated genes, indicating an even stronger transcriptional response than in DS1. The DS1 vs. DS2 comparison (Figure 2c) revealed 9413 DEGs, comprising 4639 upregulated and 4774 downregulated genes, reflecting distinct transcriptomic adjustments between the two drought-stressed stages. Due to limited sample availability and the lack of preserved RNA material, qRT-PCR validation was not conducted in this study. However, the RNA-seq data showed high consistency across biological replicates and treatment-specific clustering, supporting the reliability of the expression patterns.

### 2.3. KEGG Pathway Enrichment Analysis of Differentially Expressed Genes

**Figure 3:**
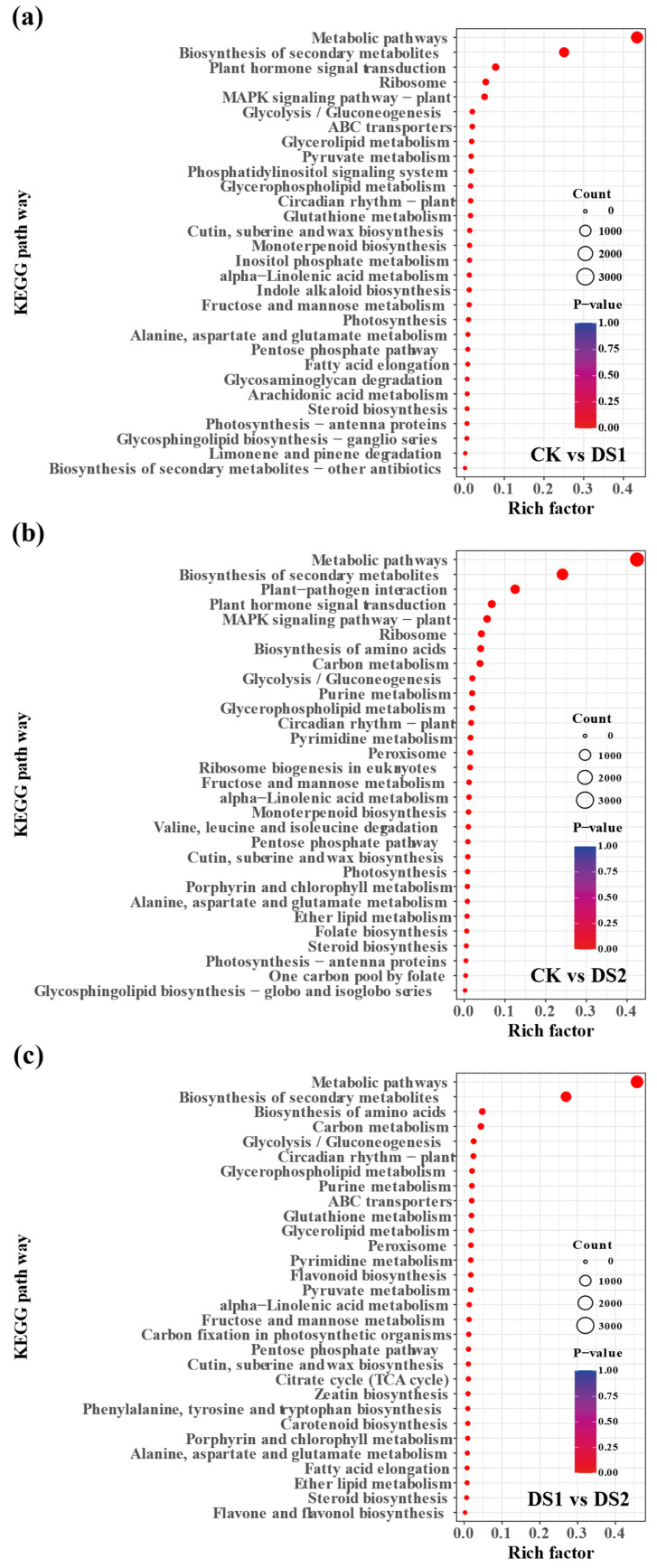
Kyoto Encyclopedia of Genes and Genomes (KEGG) pathway enrichment analysis of significantly differentially expressed genes (DEGs) in sweet potato (*Ipomoea batatas* L.) leaves under drought stress at different developmental stages. Shown are the top 30 enriched KEGG pathways identified from the following pairwise comparisons: (**a**) CK (control; soil moisture maintained at 75 ± 5% field capacity (FC) throughout the growth period) vs. DS1 (drought stress; soil moisture maintained at 45 ± 5% FC for 15 days during the branching and tuber formation stage, 55–70 days after planting); (**b**) CK vs. DS2 (drought stress; soil moisture maintained at 45 ± 5% FC for 15 days during the storage root expansion stage, 90–105 days after planting); and (**c**) DS1 vs. DS2. The y–axis indicates the KEGG pathways, and the x–axis represents the Rich factor (the ratio of DEGs to the total number of annotated genes in each pathway). Dot size reflects the number of DEGs involved in each pathway, while dot color indicates the significance level of pathway enrichment (the darker the color, the smaller the *p*-value).

To explore the biological functions of the DEGs, KEGG pathway enrichment analysis was conducted (Figure 3). In CK vs. DS1 (Figure 3a), the most significantly enriched pathways included metabolic pathways, biosynthesis of secondary metabolites, plant hormone signal transduction, and the MAPK signaling pathway, indicating the involvement of primary and secondary metabolism, hormonal regulation, and stress signaling. Pathways related to ribosome function, glycolysis/gluconeogenesis, and glutathione metabolism were also enriched, reflecting oxidative stress defense and energy metabolism adjustments.

A similar pattern was observed in the CK vs. DS2 comparison (Figure 3b), with additional enrichment of amino acid biosynthesis, pyrimidine metabolism, and peroxisome-related pathways, suggesting more extensive impacts on nitrogen metabolism, nucleotide synthesis, and ROS detoxification. The enrichment of photosynthesis-related pathways implies that drought stress may impair carbon assimilation at this stage.

In the DS1 vs. DS2 comparison (Figure 3c), flavonoid biosynthesis, phenylalanine, tyrosine, and tryptophan biosynthesis, zeatin biosynthesis, and carotenoid biosynthesis were significantly enriched, highlighting stage-specific regulation of flavonoid metabolism and hormonal responses.

### 2.4. Metabolomic Analysis of Sweet Potato Leaves Under Drought Stress

**Figure 4:**
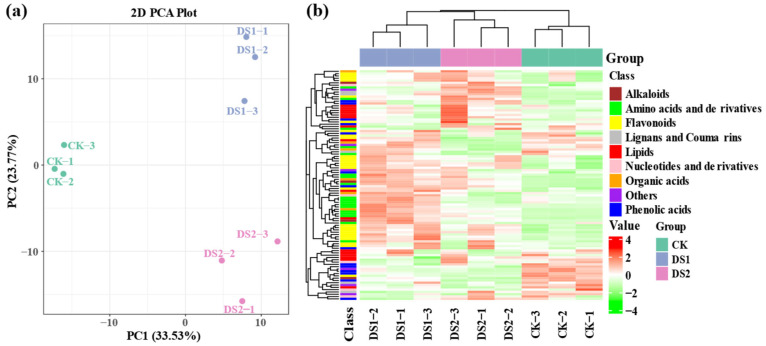
Metabolomic analysis of sweet potato (*Ipomoea batatas* L.) leaves under drought stress at different growth stages. (**a**) Principal component analysis (PCA) plot illustrating the separation of metabolic profiles among treatment groups. (**b**) Hierarchical clustering heatmap of significantly differentially accumulated metabolites (DAMs). Each row represents a metabolite, categorized by chemical class as indicated by the color bars on the left, and each column represents an individual sample grouped by treatment. The color gradient from green (low abundance) to red (high abundance) indicates the normalized abundance (Z-score) of metabolites. CK, soil moisture maintained at 75 ± 5% field capacity (FC) throughout the growth period; DS1, drought stress with soil moisture maintained at 45 ± 5% FC for 15 days during the branching and tuber formation stage (55–70 days after planting); DS2, drought stress with soil moisture maintained at 45 ± 5% FC for 15 days during the storage root expansion stage (90–105 days after planting).

To evaluate metabolite responses to drought stress, PCA and HCA were performed (Figure 4). PCA (Figure 4a) revealed a clear separation among the three groups, indicating substantial metabolic reprogramming. PC1 and PC2 accounted for 33.53% and 23.77% of the variance, respectively. CK samples clustered tightly, whereas DS1 and DS2 formed distinct clusters, with DS2 showing a greater deviation from CK, suggesting a stronger metabolic shift during the storage root expansion stage.

The hierarchical clustering heatmap (Figure 4b) further demonstrated distinct metabolite accumulation patterns. Metabolites were categorized into classes, including flavonoids, amino acids and derivatives, phenolic acids, alkaloids, lignans and coumarins, nucleotides and derivatives, organic acids, and lipids. Flavonoids were notably upregulated in DS2, highlighting their potential role in drought adaptation during the storage root expansion stage. DS1 and DS2 exhibited distinct metabolic profiles, with DS2 showing more pronounced shifts compared to CK.

### 2.5. Classification and Distribution of Differentially Accumulated Metabolites

**Figure 5:**
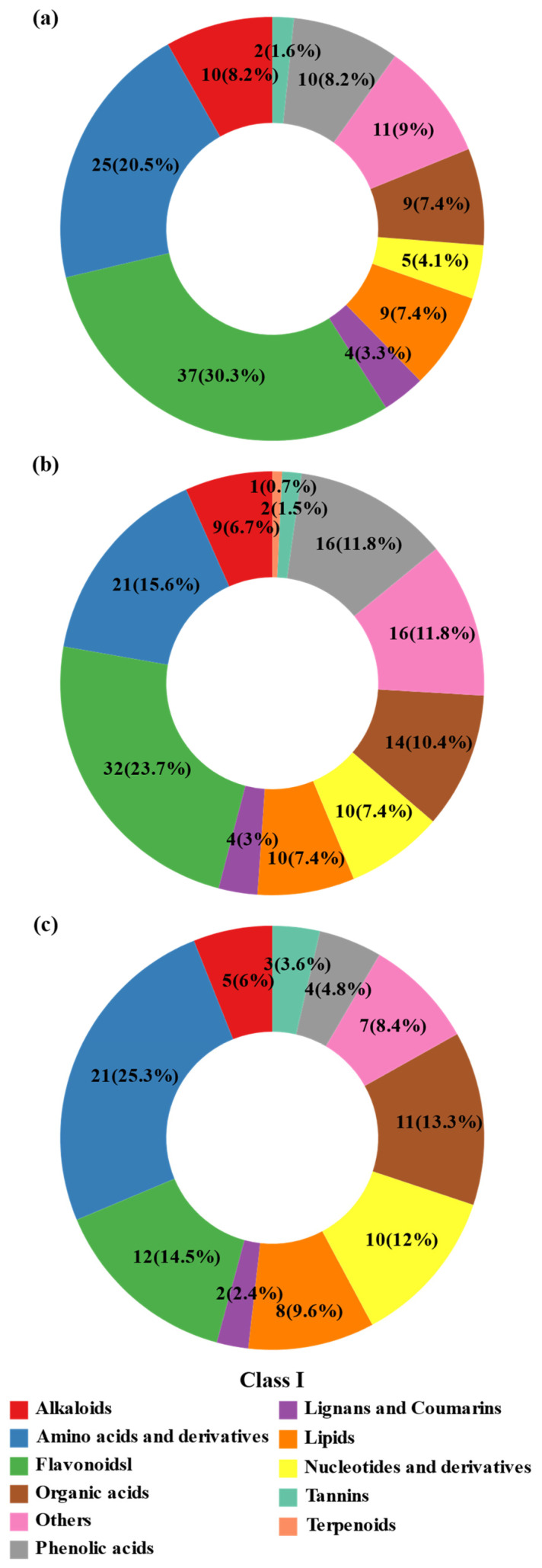
Classification and distribution of significantly differentially accumulated metabolites (DAMs) identified in sweet potato (*Ipomoea batatas* L.) leaves under drought stress at different growth stages. The composition of DAMs is shown for pairwise comparisons among treatments: (**a**) CK (control; soil moisture maintained at 75 ± 5% field capacity (FC) throughout the growth period) vs. DS1 (drought stress; soil moisture maintained at 45 ± 5% FC for 15 days during the branching and tuber formation stage, 55–70 days after planting); (**b**) CK vs. DS2 (drought stress; soil moisture maintained at 45 ± 5% FC for 15 days during the storage root expansion stage, 90–105 days after planting); and (**c**) DS1 vs. DS2.

Significant differential metabolites were classified into biochemical categories and visualized using donut charts (Figure 5). In the CK vs. DS1 comparison (Figure 5a), flavonoids accounted for 30.3% of differential metabolites, followed by amino acids and derivatives (20.5%), alkaloids (8.2%), phenolic acids (8.2%), organic acids (7.4%), lipids (7.4%) nucleotides and derivatives (4.1%), lignans and coumarins (3.3%), and tannins (1.6%).

In CK vs. DS2 (Figure 5b), flavonoids remained dominant (23.7%), although their relative abundance decreased slightly compared to DS1. Amino acids and derivatives (15.6%), organic acids (10.4%), nucleotides and derivatives (7.4%), and lipids (7.4%) remained abundant. The proportion of metabolites classified as “others” increased (11.9%), suggesting broader metabolic reprogramming during the storage root expansion stage.

In DS1 vs. DS2 (Figure 5c), amino acids and derivatives became the most prominent class (25.3%), followed by flavonoids (14.5%), organic acids (13.3%), and nucleotides and derivatives (12%). Notably, the proportion of terpenoids (9.6%) and phenolic acids (4.8%) also increased compared to DS1, suggesting their potential involvement in stage-specific drought adaptation mechanisms.

### 2.6. Identification of Differentially Accumulated Metabolites Under Drought Stress

**Figure 6:**
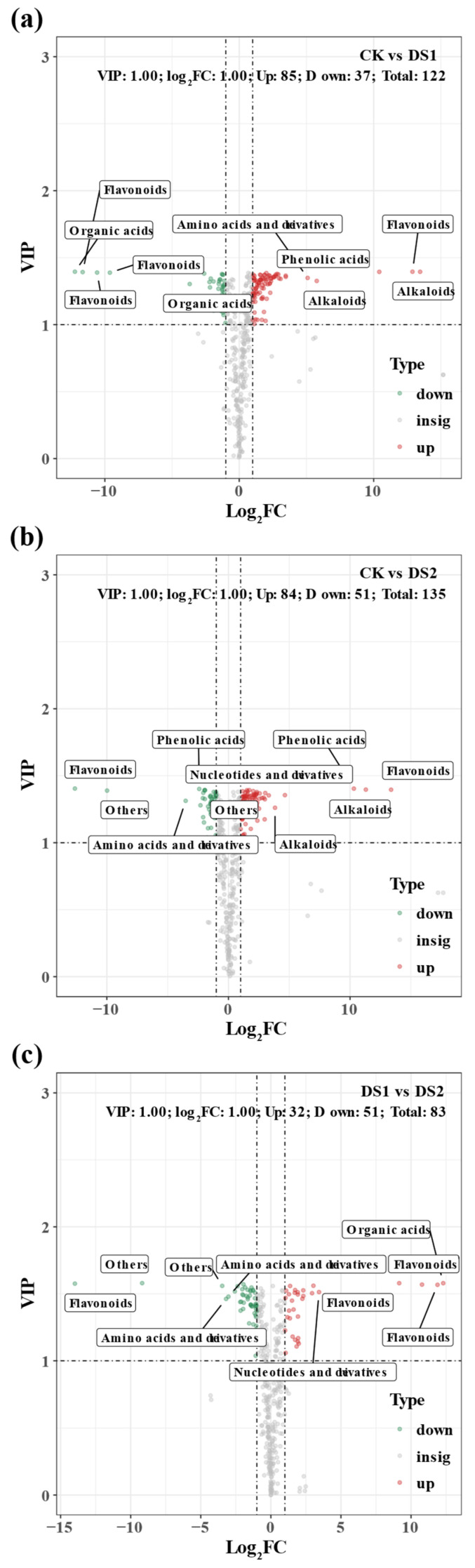
Volcano plots showing differentially accumulated metabolites (DAMs) in sweet potato (*Ipomoea batatas* L.) leaves under drought stress at different growth stages. Pairwise comparisons of metabolite profiles among treatments: (**a**) CK (control; soil moisture maintained at 75 ± 5% field capacity (FC) throughout the growth period) vs. DS1 (drought stress; soil moisture maintained at 45 ± 5% FC for 15 days during the branching and tuber formation stage, 55–70 days after planting); (**b**) CK vs. DS2 (drought stress; soil moisture maintained at 45 ± 5% FC for 15 days during the storage root expansion stage, 90–105 days after planting); and (**c**) DS1 vs. DS2. Horizontal and vertical dashed lines indicate significance thresholds (|log_2_ fold change| ≥ 1 and VIP ≥ 1). Red and green dots represent significantly upregulated and downregulated metabolites, respectively, while gray dots denote non-significant metabolites. The top five metabolite classes with the highest numbers of up- and down-regulated metabolites are annotated.

Volcano plots were generated to visualize DAMs across treatments (Figure 6). In CK vs. DS1 (Figure 6a), 122 DAMs were identified, with 85 upregulated and 37 downregulated, notably including flavonoids, organic acids, amino acids and derivatives, and phenolic acids.

In CK vs. DS2 (Figure 6b), 135 DAMs were detected, with 84 upregulated and 51 downregulated. Flavonoids, nucleotides and derivatives, alkaloids, and phenolic acids showed increased accumulation, indicating broader metabolic reprogramming during the storage root expansion stage.

In DS1 vs. DS2 (Figure 6c), 32 DAMs were upregulated and 51 downregulated. Amino acids and derivatives showed strong upregulation in DS2 compared to DS1. Organic acids, nucleotides, and other secondary metabolites also exhibited significant changes, suggesting that metabolic regulation is growth-stage dependent.

### 2.7. KEGG Enrichment Analysis of Differentially Accumulated Metabolites

**Figure 7:**
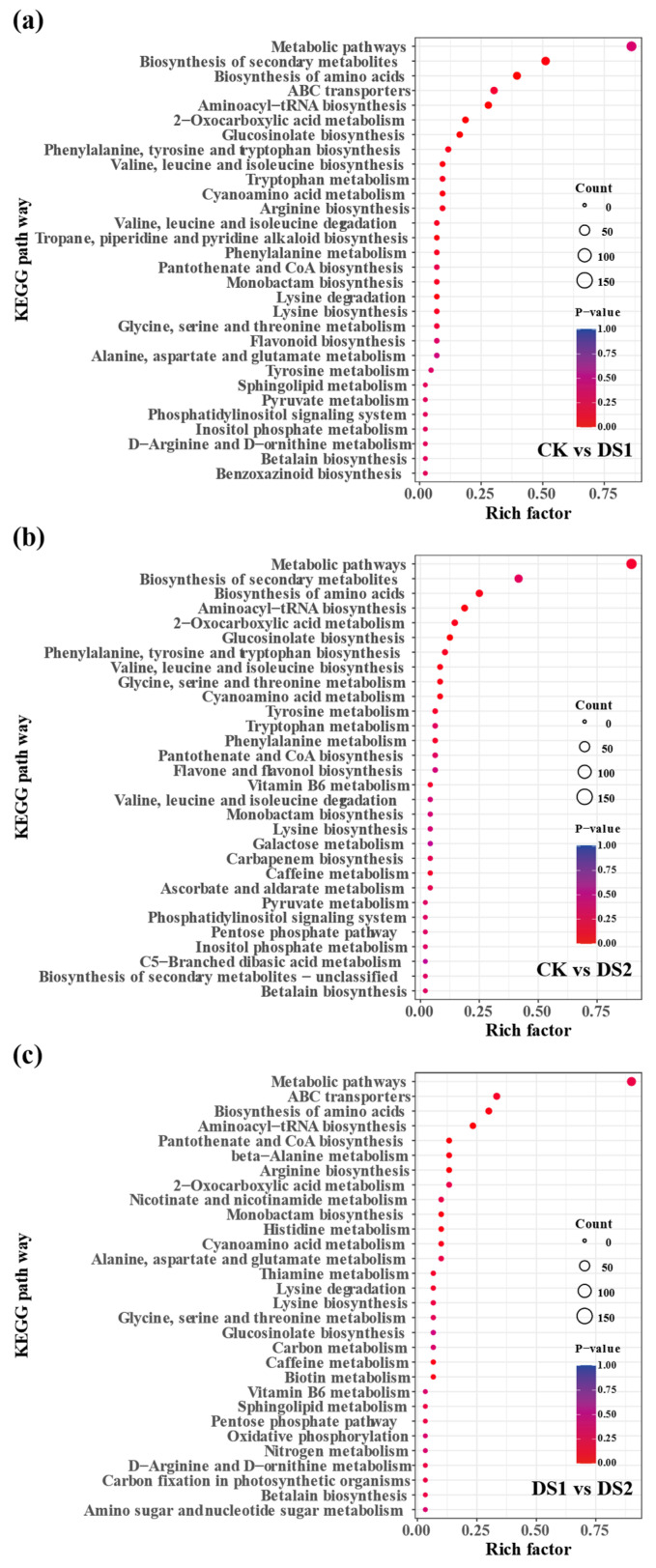
KEGG pathway enrichment analysis of differentially accumulated metabolites (DAMs) in sweet potato (*Ipomoea batatas* L.) leaves under drought stress at different growth stages. The top 30 enriched KEGG metabolic pathways identified from pairwise comparisons are shown: (**a**) CK (control; soil moisture maintained at 75 ± 5% field capacity (FC) throughout the growth period) vs. DS1 (drought stress; soil moisture maintained at 45 ± 5% FC for 15 days during the branching and tuber formation stage, 55–70 days after planting); (**b**) CK vs. DS2 (drought stress; soil moisture maintained at 45 ± 5% FC for 15 days during the storage root expansion stage, 90–105 days after planting); and (**c**) DS1 vs. DS2. The x–axis represents the Rich factor (the proportion of DAMs in each pathway relative to the total annotated metabolites). Dot size reflects the number of significantly enriched DAMs, while dot color indicates statistical significance, with red representing higher significance (smaller *p*-values).

KEGG enrichment analysis of DAMs revealed significant metabolic pathways affected by drought stress (Figure 7). In CK vs. DS1 (Figure 7a), key pathways included biosynthesis of secondary metabolites, amino acid biosynthesis, ABC transporters, aminoacyl-tRNA biosynthesis, phenylalanine, tyrosine, and tryptophan biosynthesis, and flavonoid biosynthesis. Pathways related to glutathione metabolism and nitrogen-containing secondary metabolites were also enriched, indicating oxidative stress responses.

In CK vs. DS2 (Figure 7b), similar pathways were enriched, with additional enrichment of pantothenate and CoA biosynthesis, vitamin B6 metabolism, and galactose metabolism, suggesting broader metabolic reprogramming. Flavonoid biosynthesis remained significantly enriched.

In DS1 vs. DS2 (Figure 7c), amino acid biosynthesis, ABC transporters, oxidative phosphorylation, carbon metabolism, and thiamine metabolism were enriched. The pentose phosphate pathway was also significantly enriched, indicating shifts in energy metabolism. Flavonoid biosynthesis remained enriched, reinforcing its pivotal role.

### 2.8. Integrated Transcriptomic and Metabolomic Analysis of Flavonoid and Flavonol Biosynthesis Pathways

**Figure 8:**
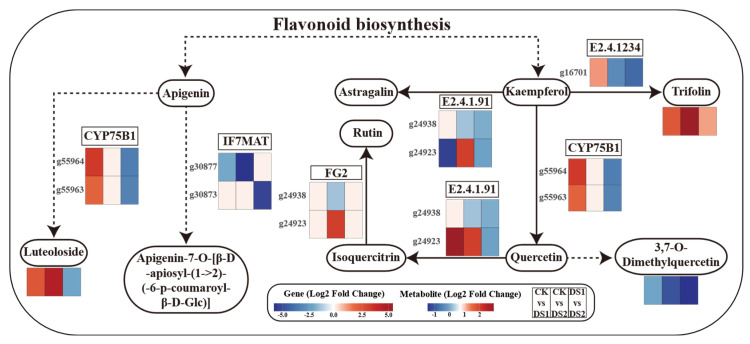
Integrated transcriptomic and metabolomic analysis of flavone and flavonol biosynthesis pathways in sweet potato (*Ipomoea batatas* L.) leaves under drought stress at different growth stages. Heatmaps show the log_2_ fold changes of gene expression (squares) and metabolite abundance (ovals) derived from transcriptomic and metabolomic data in the following pairwise comparisons: CK (control; soil moisture maintained at 75 ± 5% field capacity (FC) throughout the growth period); DS1 (drought stress; soil moisture maintained at 45 ± 5% FC for 15 days during the branching and tuber formation stage, 55–70 days after planting); and DS2 (drought stress; soil moisture maintained at 45 ± 5% FC for 15 days during the storage root expansion stage, 90–105 days after planting).

An integrated analysis was conducted on flavone and flavonol biosynthesis pathways (Figure 8). *CYP75B1*, involved in flavonoid hydroxylation, and *IF7MAT*, related to flavone glycosylation, were strongly downregulated under drought stress. Flavonol synthase and glycosyltransferases genes exhibited stage-specific regulation, suggesting growth-stage-dependent modulation of flavonoid biosynthesis.

At the metabolite level, quercetin, isoquercitrin, and rutin were significantly downregulated, particularly in DS2, suggesting sensitivity to prolonged drought. Conversely, trifolin and luteoloside were upregulated in both DS1 and DS2, indicating their potential protective roles.

Overall, the integrated analysis revealed a complex regulatory network governing flavonoid metabolism under drought stress, with distinct molecular adjustments between DS1 and DS2. Early-stage drought stress appeared to favor the accumulation of specific flavone glycosides, whereas prolonged stress led to broader suppression of flavonoids, potentially affecting stress-related signaling and antioxidant defense.

## 3. Discussion

### 3.1. Stage-Specific Transcriptomic Responses to Drought Stress

Sweet potato, as a shallow-rooted crop, is highly susceptible to water deficits, especially during critical growth periods such as the branching and tuber formation stage and the storage root expansion stage [38]. In this study, significant clustering of CK, DS1, and DS2 groups in PCA and HCA (Figure 1a,b) emphasized the profound transcriptional changes induced by drought stress. Notably, a substantially higher number of DEGs were identified in DS2 (13,509) compared to DS1 (8292), highlighting more pronounced transcriptional reprogramming during the storage root expansion stage. This finding aligns with previous studies showing that late-stage drought stress has a greater physiological impact on sweet potato, particularly affecting storage root development and carbon allocation [39,40,41].

KEGG enrichment analysis revealed that both DS1 and DS2 activated key drought-responsive pathways, including phytohormone signaling, secondary metabolite biosynthesis, glutathione metabolism, and carbon metabolism. However, comparative analysis indicated that flavonoid and carotenoid biosynthesis pathways were specifically enriched in DS2, supporting stage-specific metabolic regulation [37,42,43]. These differences suggest that drought stress influences not only stress-responsive genes but also basal metabolic and developmental pathways in a stage-dependent manner [44].

The enhanced transcriptional plasticity observed during DS2 may reflect the plant’s intensified efforts to sustain carbon assimilation and osmotic homeostasis during the root expansion phase, which is characterized by elevated energy demands [45]. This phenomenon may partially explain the 30–50% yield loss reported in field studies under late-season drought [46,47]. In contrast, metabolic responses at the DS1 stage were more targeted, especially the accumulation of specific flavonoids, suggesting that early defense mechanisms prioritize rapid ROS scavenging and the stabilization of leaf metabolism [48]. These findings are consistent with the shallow-rooted nature of sweet potato, which amplifies its vulnerability to water deficits and necessitates distinct regulatory mechanisms throughout its ontogeny [49].

### 3.2. Metabolic Reprogramming Under Drought Stress

The metabolomic analysis revealed that drought stress induced pronounced and stage-specific metabolic alterations. PCA results showed clear separation among CK, DS1, and DS2 groups, with DS2 exhibiting a greater deviation (PC1 + PC2: 57.30%), consistent with transcriptomic patterns. Flavonoids were the predominant class of DAMs during both DS1 (30.3%) and DS2 (23.7%), underscoring their pivotal role in drought stress responses. Flavonoids, renowned for their antioxidant properties [50], may alleviate drought-induced oxidative stress by scavenging ROS generated from impaired photosynthetic activity [51]. This is corroborated by the enrichment of flavonoid biosynthesis pathways, particularly in DS1 vs. DS2 (Figure 3c), suggesting that metabolic reprogramming during DS2 enhances ROS detoxification [52]. In DS1, 293 of the 415 DAMs were upregulated, including flavonoids, amino acids, and organic acids, likely supporting osmoregulation and antioxidant defense [53]. In DS2, 280 upregulated DAMs were mainly involved in nucleotide, alkaloid, and coenzyme metabolism (e.g., pantothenate biosynthesis), reflecting sustained stress adaptation and carbon redistribution to tubers [54]. These findings align with reports suggesting that late-stage drought exerts a more severe physiological impact on sweet potato by disrupting storage root development and carbon allocation [55,56]. The metabolic characteristics of these stages suggest that sweet potato may adopt a dual drought response strategy: an early defense surge followed by a sustained adaptation phase.

### 3.3. Coordinated Regulation of Flavonoid Biosynthesis: An Integrated Omics Perspective

Integration of transcriptomic and metabolomic data indicated that flavonoid biosynthesis plays a central role in the sweet potato drought stress response. Combined analysis revealed that although key flavonoid biosynthetic genes such as *CYP75B1* and *IF7MAT* were downregulated in both DS1 and DS2, genes encoding flavonol synthase and glycosyltransferases displayed stage-specific transcriptional regulation, potentially contributing to differential flavonoid accumulation, including trifolirhizin, baicalin, quercetin, and rutin [57].

Under drought stress, especially during DS1, the accumulation of tricin and luteolin was elevated, suggesting their involvement in early defense responses through enhanced ROS scavenging [58]. Conversely, quercetin and rutin levels decreased during DS2, indicating that specific branches of the flavonoid biosynthesis pathway were selectively downregulated, possibly to redirect metabolic flux toward alternative protective compounds or to conserve energy under prolonged drought stress [59].

Across all comparisons, flavonoids exhibited consistent enrichment, with phase-specific patterns indicating an early defense role during DS1 and a resource conservation role during DS2. These coordinated changes suggest that flavonoid metabolism not only mediates oxidative stress responses but also contributes to the developmental trade-off between growth and defense. This dual role highlights the potential of flavonoids as biomarkers for drought tolerance [60].

Furthermore, the expression trends of key genes such as *CYP75B1* and *IF7MAT* observed in our study are consistent with previous transcriptomic and metabolomic reports in sweet potato and related species [22,36,37]. Notably, recent work in *Sophora alopecuroides* revealed that *IF7MAT* expression was significantly downregulated under moderate drought stress, in parallel with reduced flavonoid accumulation, supporting a conserved role for this gene in drought-related metabolic regulation [61].

## 4. Materials and Methods

### 4.1. Plant Material and Stress Treatment

The sweet potato cultivar “Luoshu 11”, selected for its regional agricultural significance, was grown in a rainout shelter using a completely randomized block design with three replicates. Plants were cultivated in pots (50 cm in diameter and 60 cm in depth) filled with air-dried loamy soil collected from the experimental station field. Uniform fertilization was applied using a compound NPK fertilizer (15:15:15) at a rate of 30 g per pot. Three treatments were applied: (1) CK, with soil moisture maintained at 75 ± 5% FC throughout the growth period; (2) DS1, with soil moisture maintained at 45 ± 5% FC for 15 days during the branching and tuber formation stage (55–70 days post-planting); and (3) DS2, with soil moisture maintained at 45 ± 5% FC for 15 days during the storage root expansion stage (90–105 days post-planting). Drought stress was applied by withholding irrigation and maintaining soil moisture at 45 ± 5% of FC. Soil moisture was monitored daily using an HH2 moisture meter (Delta-T Devices, Cambridge, UK), and water was supplemented as needed to maintain the target moisture level. As the soil had been pre-air-dried, the initial moisture content was very low, allowing for accurate and consistent control of the drought stress conditions. At the end of the drought stress treatments, the third fully expanded leaf from the shoot apex of each plant was collected. For each treatment, leaves from three randomly selected plants were pooled to form one composite sample, with three biological replicates per treatment. Each composite sample was evenly divided into two subsamples, immediately placed into 15 mL collection tubes, flash-frozen in liquid nitrogen, and stored at −80 °C until further analysis. One subsample was used for transcriptomic sequencing, and the other for broad-targeted metabolomic analysis.

### 4.2. Widely Targeted Metabolomics Analysis

#### 4.2.1. Sample Preparation and Metabolite Extraction

Sweet potato leaf samples were freeze-dried (~48 h) using a vacuum lyophilizer and ground into a fine powder (MM 400 mixer mill, 30 Hz, 1–1.5 min). For extraction, 50 mg of powder was mixed with 1.2 mL of 70% (*v*/*v*) pre-chilled methanol (−20 °C), vortexed every 30 min for 30 s, and incubated overnight at 4 °C. After centrifugation (12,000 rpm, 10 min, 4 °C), the supernatant was filtered through a 0.22 μm PTFE membrane prior to UPLC-MS/MS analysis. Pooled quality control (QC) samples were injected periodically to assess instrument stability [62].

#### 4.2.2. UPLC-MS/MS Analysis

Metabolomic profiling was performed using a UPLC-MS/MS system (Shim-pack UFLC SHIMADZU CBM30A) coupled to a QTRAP 4500 mass spectrometer (SCIEX). Separation was conducted on an Agilent SB-C18 column (1.8 μm, 2.1 mm× 100 mm) maintained at 40 °C. The mobile phases were solvent A (0.1% formic acid in water) and solvent B (0.1% formic acid in acetonitrile) with a binary gradient: 95% A/5% B at 0 min; 95% B at 9 min; 5% B at 11.1 min; and re-equilibration at 14 min. The flow rate was 0.35 mL/min, and the injection volume was 2–4 μL [63].

#### 4.2.3. Mass Spectrometry Parameters

The ESI source operated in both positive and negative ionization modes with a source temperature of 500–550 °C. Ion spray voltage was set at +5500 V (positive) and −4500 V (negative). Nitrogen served as the nebulizing and auxiliary gas (curtain gas: 25–30 psi; Gas 1: 50 psi; Gas 2: 60 psi). Collision-activated dissociation (CAD) gas was set to high. The mass spectrometer was tuned with polypropylene glycol solutions, and multiple reaction monitoring (MRM) mode was used for data acquisition (Analyst 1.6.3, SCIEX). Each metabolite was monitored by two fragment ion transitions, with declustering potential (DP) and collision energy (CE) optimized via standard solution infusion.

#### 4.2.4. Metabolite Identification and Quantification

Metabolites were identified by matching MS/MS spectra to an in-house library, the Human Metabolome Database (HMDB), and the KEGG Compound database based on mass spectra, retention time, and spectral similarity scores. Authentic standards, purchased from Merck (Darmstadt, Germany), BioBioPha (Kunming, China), and Sigma-Aldrich (St. Louis, MO, USA), were used when available. Common adducts (Na^+^, K^+^, NH_4_^+^) were excluded to avoid redundancy. Internal standards, included as part of a proprietary mixture provided by the analytical platform (Metware Biotechnology, Wuhan, China), were added to each sample to monitor extraction efficiency and instrument stability. Additionally, polypropylene glycol solutions (10 and 100 μmol/L) were used for mass spectrometry calibration in both QQQ and LIT modes. Quantification was performed using MRM peak areas normalized to sample dry weight (50 mg) [64].

#### 4.2.5. Statistical Analysis of Metabolomic Data

Raw MRM peak areas were log_2_-transformed and auto-scaled (mean-centered, unit variance normalized). PCA was used to assess sample clustering, followed by orthogonal partial least-squares discriminant analysis (OPLS-DA) for discriminating metabolite patterns. Model validity was evaluated using 200-fold permutation tests. Metabolites were considered differentially accumulated if VIP ≥ 1.0, |log_2_FC| ≥ 1, and *p* < 0.05 [65,66].

#### 4.2.6. Metabolic Pathway Analysis

Differential metabolites were mapped to KEGG pathways using KEGG Compound IDs. Metabolite set enrichment analysis (MSEA) was performed using hypergeometric tests, with *p* < 0.05 considered significant [67].

### 4.3. Transcriptome Sequencing and Data Analysis

#### 4.3.1. RNA Extraction and Quality Assessment

Total RNA was extracted using the TRIzol reagent (Invitrogen, Carlsbad, CA, USA) following the manufacturer’s protocol. RNA purity and concentration were assessed using a NanoPhotometer (IMPLEN, Munich, Germany) and a Qubit 4.0 fluorometer (Thermo Fisher Scientific, Waltham, MA, USA), respectively. RNA integrity was verified by agarose gel electrophoresis and an Agilent 2100 Bioanalyzer (Agilent Technologies, Santa Clara, CA, USA), ensuring that the RNA integrity number (RIN) > 7.0 for all samples.

#### 4.3.2. Library Construction and RNA Sequencing

mRNA was enriched using oligo(dT) magnetic beads and fragmented into short sequences. First-strand cDNA synthesis was performed using random hexamer primers, followed by second-strand synthesis using dNTPs, DNA polymerase I, and RNase H. The resulting double-stranded cDNA was purified using AMPure XP beads (Beckman Coulter, Brea, CA, USA), end-repaired, A-tailed, and ligated to sequencing adapters. Libraries were validated using an Agilent 2100 Bioanalyzer (Agilent Technologies, Santa Clara, CA, USA)and qPCR to ensure insert sizes of 250–300 bp and an effective concentration > 2 nM prior to sequencing. Sequencing was conducted on an Illumina NovaSeq 6000 platform (Illumina, San Diego, CA, USA), generating 150-bp paired-end reads [68].

#### 4.3.3. Data Processing and Quality Control

Raw reads were filtered to remove adapter sequences, low-quality reads (Q < 20 in >50% of reads), and reads containing >10% N bases using FastQC (v0.11.9) and Trimmomatic (v0.39) [69]. Clean reads were aligned to the sweet potato reference genome using HISAT2 (v2.2.1) with the default parameters. Aligned reads were used for subsequent expression analyses.

#### 4.3.4. Gene Expression Quantification and Differential Expression Analysis

Gene expression levels were quantified using featureCounts (v2.0.1) and normalized using the FPKM (fragments per kilobase of transcript per million mapped reads) method. DEGs were identified using DESeq2 (v1.30.1) with thresholds of |log_2_FC| ≥ 1 and FDR < 0.05 [70].

#### 4.3.5. Functional Annotation and Enrichment Analysis

DEGs were annotated using NR, SwissProt, KEGG, KOG, Pfam, and GO databases via BLASTX (E-value < 1 × 10^−5^). KEGG pathway enrichment analysis was performed using KOBAS (v3.0), and pathways with *p* < 0.05 were considered significantly enriched [71].

### 4.4. Integrating Transcriptome and Metabolome Analysis

Significant DEGs and DAMs were jointly mapped to the KEGG database to explore functional relationships. Integrated pathway analysis was performed to visualize overlapping biological processes and molecular functions. Genes and metabolites were assigned to specific metabolic pathways based on KEGG annotations, with upregulated and downregulated elements distinguished by color coding (red: upregulated, green: downregulated, blue: mixed regulation).

### 4.5. Metabolome and Transcriptome Data Visualization

#### 4.5.1. Principal Component Analysis

PCA was performed separately for metabolomic and transcriptomic datasets to evaluate sample clustering. The first two principal components (PC1 and PC2) were extracted using FactoMineR and factoextra and visualized using ggplot2. Sample labels were adjusted with ggrepel, and treatment groups were color-coded.

#### 4.5.2. Heatmap Visualization

HCA were constructed for the top 100 DAMs and DEGs based on variance. Data were Z-score normalized and clustered using k-means (k = 9) with Euclidean distance. Heatmaps were generated using ComplexHeatmap, with annotations indicating treatment groups and cluster information.

#### 4.5.3. Metabolite Class Distribution

Donut charts were created to display metabolite classification using ggplot2. Different metabolite classes were assigned colors with RColorBrewer, and percentage labels indicated their relative proportions.

#### 4.5.4. Volcano Plots

Volcano plots were generated for metabolomic and transcriptomic comparisons (CK vs. DS1, CK vs. DS2, DS1 vs. DS2) using ggplot2. DAMs were plotted using log_2_FC and VIP values, while DEGs were plotted using log_2_FC and −log_10_ (*p*-values). Significant metabolites and genes were highlighted, and the top 10 were labeled using ggrepel. Plots were combined using patchwork.

#### 4.5.5. KEGG Pathway Enrichment Analysis

The top 30 enriched KEGG pathways were visualized using ggplot2. The Rich factor was plotted on the x–axis, and pathway names were on the y–axis. Dot size indicated the number of enriched genes or metabolites, and color represented statistical significance (*p*-value). Plots were combined using patchwork.

## 5. Conclusions

In this study, we integrated transcriptomic and metabolomic analyses to elucidate the stage-specific drought responses of the sweet potato cultivar ‘Luoyu 11’ during the branching and tuber initiation stage (DS1) and the storage root expansion stage (DS2). Drought stress induced significant molecular reprogramming at both stages, with DS2 exhibiting more extensive transcriptomic alterations and metabolic remodeling. Flavonoids play a pivotal role throughout both stages, and the stage-specific regulation of key biosynthetic genes (*CYP75B1* and *IF7MAT*) is closely associated with the differential accumulation of flavonol derivatives. Our findings demonstrate that the selective activation of flavonoids and their biosynthetic branches constitutes a core component of the drought response in sweet potato.

In addition to the known structural genes and metabolites analyzed in this study, our integrated transcriptomic and metabolomic dataset provides a valuable resource for the discovery of novel components involved in flavonoid biosynthesis and regulation. Although our current analysis focused primarily on annotated flavonoid biosynthetic genes, the co-expression patterns, stage-specific transcriptional profiles, and metabolite associations observed suggest that additional, previously uncharacterized genes may contribute to the regulation of this pathway under drought stress. Future studies could employ weighted gene co-expression network analysis (WGCNA), promoter motif scanning, or machine learning-based inference to predict novel biosynthetic or regulatory candidates, including transcription factors beyond the canonical MYB, bHLH, and WD40 families. These efforts, complemented by functional validation, would substantially deepen our understanding of the transcriptional regulation and diversification of flavonoid metabolism in sweet potato. We anticipate that such integrative approaches will not only clarify the mechanisms of drought adaptation but also support the breeding of stress-resilient cultivars through marker-assisted selection.

## Data Availability

The original raw sequencing data (FASTQ files) were generated by a third-party sequencing service provider (Wuhan Metware Metabolic Biotechnology Co., Ltd., Wuhan, China). Unfortunately, due to an oversight, the raw files were not retrieved before the provider’s data retention period expired and are no longer accessible. However, all downstream analysis results, including normalized expression matrices, differential expression results, and functional enrichment data, have been preserved and are publicly available at the Zenodo repository: https://doi.org/10.5281/zenodo.15852415. The full data processing pipeline, including software tools and parameter settings, is described in the Methods section to ensure transparency and reproducibility.
